# Estradiol progesterone ratio on ovulation induction day: a determinant of successful pregnancy outcome after intra cytoplasmic sperm injection

**Published:** 2014-09

**Authors:** Rehana Rehman, Rakhshaan Khan, Mukhtiar Baig, Mehwish Hussain, Syeda Sadia Fatima

**Affiliations:** 1 *Bahria University Medical and Dental College, Karachi, Pakistan.*; 2 *Department of Biological and Biomedical Sciences, Faculty of Health Sciences, Medical College, The Aga Khan University, Karachi, Pakistan.*; 3 *Faculty of Medicine, Rabigh, King Abdulaziz University, Jeddah, KSA.*; 4 *Research Development, Dow University of Health Sciences, Karachi, Pakistan*

**Keywords:** *Intracytoplasmic sperm injection*, *Ovulation induction*, *Pregnancy outcome*, *Implantaion*

## Abstract

**Background:** Intracytoplasmic sperm injection (ICSI) is an advanced technique employed in assisted reproductive clinics for treatment of infertile couples. The reproductive endocrinologists try their level best to identify factors that enhance success rate after ICSI.

**Objective:** To compare estradiol progesterone ratio on ovulation induction day amongst pregnancy outcome groups following ICSI.

**Materials and Methods: **A cross sectional study was conducted on 323 couples of Assisted Reproductive Clinic in Islamabad from June 2010 till August 2011. Down regulation of females aged 18-40 years with gonadotrophin releasing hormone agonist was followed by calculated stimulation with gonadotrophin injections (COS). Oocytes pickup was done 36 hours after ovulation induction by 16G adapter and double lumen oocyte aspiration needle under general anesthesia. Oocytes were fertilized in vitro, graded and only blastocysts were transferred seven days after ovulation induction. Serum estradiol and progesterone were measured by enzyme linked immuno sorbent assay on ovulation induction day, ratio was compared in three groups of females; no conception with βhCG 5-25 mIU/ml, preclinical abortion with βhCG >25 mIU/ml and no cardiac activity on transvaginal scan and clinical pregnancy with βhCG >25mIU/ml and cardiac activity on transvaginal scan.

**Results: **Females having high estradiol/ progesterone ratio were able to achieve clinical pregnancy shown by a positive βhCG and cardiac activity on transvaginal scan. These females also had significantly high number of oocytes, endometrial thickness and implantation rate.

**Conclusion:** A high estradiol/progesterone ratio on the day of ovulation induction predicts the success of intra cytoplasmic sperm injection.

## Introduction

New life is acquired through fertilization, gastrulation, and accomplished after birth of an individual through a smooth and continuous process (1). Humans, like other organisms, transfer their unique characteristics to the next generation through the process by the fertilization of female gamete by the male gamete. “A couple is considered to be experiencing infertility if conception has not occurred after 12 months of sexual activity without the use of contraception”. It is perceived as a problem across the world in all cultures and affects about 10-15% of couples of reproductive age (2). 

The proportion of couples seeking medical treatment for infertility is estimated to be 4-17% in developing countries (3). Assisted reproduction is the scientific assistance provided to the infertile couples to achieve pregnancy and enjoy parenthood by several assisted reproductive techniques that tend to overcome natural barriers to fertilization. Amongst these procedures, intra cytoplasmic sperm injection (ICSI) is a promising micro-manipulation technique, in which fertilization is accomplished by the injection of a sperm into a single egg (4). 

Estradiol (E_2_) is a hormone produced by granulosa cells of ovaries by the aromatization of androstenedione to estrone in the follicular phase of the cycle. The E_2_ levels in ICSI procedures are subject to deviation by the widespread use of gonadotrophin releasing hormone (GnRH) agonists and antagonists for down-regulation of ovaries followed by controlled ovarian stimulation (COS) (5, 6). It has been found that peak E_2_ levels measured on the day of human chorionic gonadotrophin (hCG) administration helps in assessment of response to COS and higher peak E_2_ levels are associated with better pregnancy rates achieved (7, 8). Thus, produced E_2_ , orchestrate endometrium lining with encroaching blastocysts by a series of events that initiate hypertrophy and hyperplasia of the endometrium followed by development of progesterone (P) receptors. 

Progesterone is a hormone of secretory phase that increases uterine receptivity by various mechanisms like mast cell maturation, degranulation, production of cytokines and growth factors for blastocysts implantation, successful conception and continuation of pregnancy (9-12). It has been debated for many years whether P increases in the late follicular phase of COS has a detrimental effect on the outcome of IVF or not and reduced implantation and pregnancy rates were reported by few but not all investigators (10, 12, 13). 

Failure of treatment procedure in assisted reproductive clinics (ARC) suggests a lack of implantation due to failure of coordination between maternal and fetal interfaces. One of the factors which is responsible for inadequate preparation of endometrial bed for encroaching blastocyst is scarcity of optimal concentration of E_2_ and P (13). Research has been done to evaluate the role of E_2_/P ratio in the luteal phase however; the results are debatable (14-16). These results are subject to bias because of the use of P supplementation before estimation of E_2_/P levels. The current study is aimed to evaluate the effect of E_2_/P ratio on ovulation induction (OI) day (before P supplementation) with respect to the pregnancy outcome after ICSI in our Pakistani females.

## Materials and methods

It was a cross sectional study conducted from June 2010 till August 2011 after ethical approval from Ethical Review Board of Intracytoplasmic sperm injection @ Saudi Pak Tower. The convenience sampling technique was employed to collect the samples of 323 consented couples who strictly fulfilled our inclusion criteria. Informed written and oral consent was acquired by women included in the study with age range between 18-40 years, duration of infertility more than 2 years, intact ovaries without morphological abnormalities, normal ovulatory cycle (25-35 days), body mass index (BMI) of 18-27 kg/m^2^, basal FSH (day two) serum level <10 IU/mL, were selected for long protocol with GnRh agonist, stimulated with injection of recombinant follicle stimulating hormone (rFSH; Puregon) and kept on P support with 400 mg cyclogest pessaries. Females on GnRh antagonist, short down regulation with GnRh agonist and ICSI with sperm retrieval by testicular biopsy were excluded from the study.

Selected women were down regulated with daily injection deca peptyl (GnRh agonist) from day 21 of previous cycle followed by COS with rFSH; Injection Puregon S/C from second or third day of cycle for fourteen days. Maturity of follicle was assessed by series of transvaginal scan (TVS) started from the fifth day of COS till OI with intra muscular injection of human chorionic gonadotrophin (hCG; Pregnyl 10,000 I.U). The venous samples were taken for estimation of peak E_2_ and P on this day. Oocyte pick up (OPU) was performed 36 hours after OI (COS±14 days) by which oocytes were retrieved by vaginal ultrasound probe with 16G adapter and double lumen oocyte aspiration needle. Collected oocytes were treated and then transferred to the incubator for about 1-2 hours prior to insemination by ICSI procedures. 

Semen analysis was performed by strict Kruger’s criteria with impaired male fertility potential considered with less than 4% normal sperm morphology (17). ICSI was performed by micro injections of spermatozoa at right angles to the position of polar body under the microscope. Fertilized embryos (presence of two pronuclei; 2PN) were assessed and graded daily for their developmental characteristics in vitro; cleavage till differentiation into distinct cell types with formation of fluid filled cavity (blastocysts). Embryo transfer (ET) of blastocysts was done seven days after OI by Sims-Wallace embryo replacement catheter under ultrasound guidance. Luteal support was maintained by P vaginal pessaries (Cyclogest 400 mg) twice a day from the day of OPU. 

**Outcome measures**

Single serum beta hCG measurement was performed on specimens obtained by peripheral venipuncture 14 days after OPU as the outcome marker. TVS was performed at five weeks of gestation to detect clinical pregnancy and differentiate it from preclinical abortion. On the basis of beta hCG and TVS, results were grouped as: non pregnant females with beta hCG 5-25 mIU/ml were categorized into group I; women with preclinical abortion and beta hCG >25 m IU/ml and no fetal cardiac activity on TVS were group II; while women with clinical pregnancy (CP) with beta hCG >25 mIU/ml and cardiac activity confirmed by TVS were labeled as group III (15). The E_2_/P ratio was calculated in all three groups. Fertilization rate was defined as “the proportion of oocytes resulting in two pronuclei formation” (11). Mean implantation rate was the proportion of embryos transferred resulting in an intrauterine gestational sac. A clinical pregnancy was defined by the presence of one or more gestation sacs by ultrasound (18).

**Statistical analysis**

Data was analyzed using SPSS software (Statistical Package for the Social Sciences, version 16.0, SPSS Inc, Chicago, Illinois, USA). Shapiro-Wilk’s test was used to check normality of continuous variables. Mean±SD was computed to present normally distributed continuous variables. Comparison of these variables among groups was tested by one way Analysis of Variance (ANOVA). Median (Interquartile range) was computed to present skewed variables. Kruskal Wallis test was run to compare skewed variables among different groups of pregnancy outcomes. Frequencies and percentages were computed for categorical variables. 

Chi-square test was executed to compare the categorical variables among pregnancy outcomes. Level of significance was set equal to 5%. To ensure prediction accuracy of E_2_, P and the ratio of E_2_/P for pregnancy outcome, receiver operating characteristics (ROC) curve was plotted via Med Calc Software (version 12.7.3.0). For the same analysis, CP group was taken as state value and compared with preclinical abortion. 

## Results

**Figure 1:**
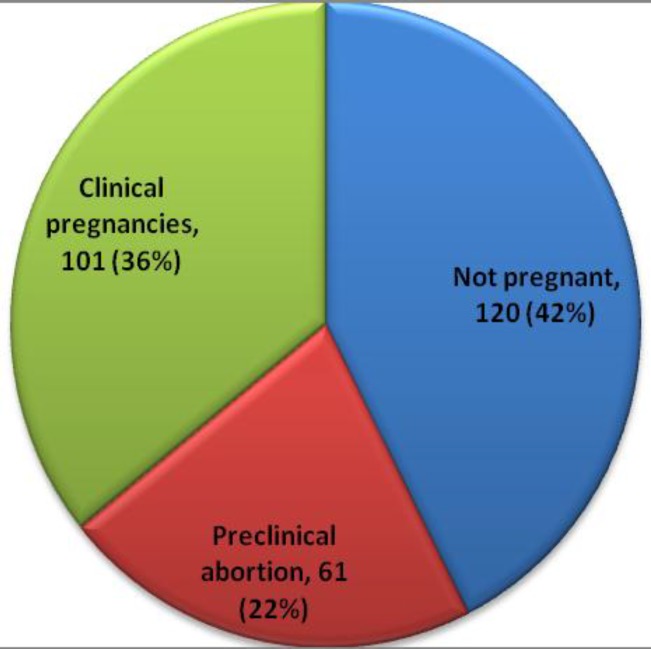
Distribution of patients on the basis of outcome after ICSI

**Figure 2:**
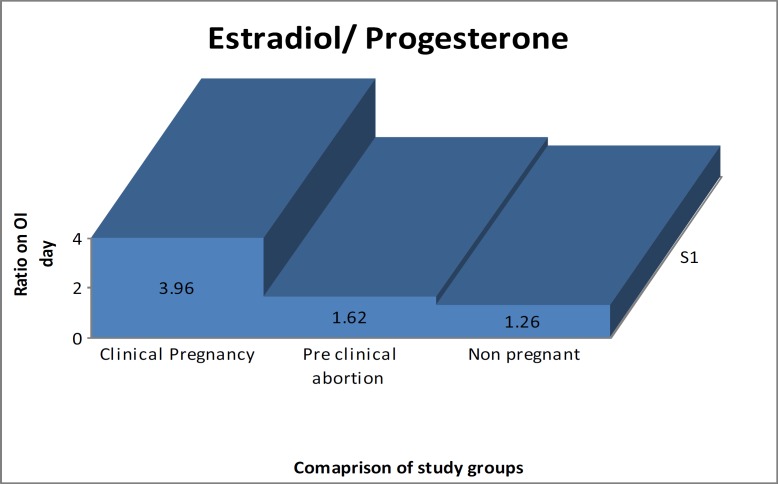
Estradiol/progesterone ratio on the day of ovulation induction

Out of 323 recruited patients, ET was carried out in 282 patients (87.30%) due to inadequate ovarian response in 14 patients (4.33%), and embryos transferred before blastocyst maturation in 27 patients (8.35%). Among these 282 females, a high E_2_/P ratio was seen in 101 (36%) patients who had clinical pregnancy (group III), 61 (22%) had lower E_2_/P ratio and presented with preclinical abortion (group II), 120 (42%) did not achieve pregnancy (Group I) as given in figure 1. The E_2_/P ratio is significantly high in women in the CP group (p<0.001) as shown in figure 2.

Demographic data presented in table I demonstrates that ages at marriage and at the time of booking were almost the same in all three groups, however, BMI was higher in females who failed to achieve pregnancy (p=0.028). The numbers of oocytes at different phases were observed significantly more in women with high E_2_/P (p<0.0001). Comparison of P levels on basal (day 2 of the previous cycle) and OI day reveals that CP group with high E_2_/P had high basal and low P levels on OI day. Fertilization rate was found to be the same in all groups (p=0.203) while implantation rate was more in CP group (p<0.0001) with high E_2_/P (Table II).

FSH was found more in non-pregnant females with low E_2_/P. Basal E_2_ was less in these females (p<0.001) and numbers of puregons were found significantly more in non-pregnant group. Leutinising hormone (LH) was highest in the CP group with high E_2_/P while Prolactin was the same in all three groups. Endometrial thickness was found to be quite low in females who failed to achieve pregnancy (non-pregnant group). The statistics of the ovarian response to COS are displayed in table III.

**Figure 3:**
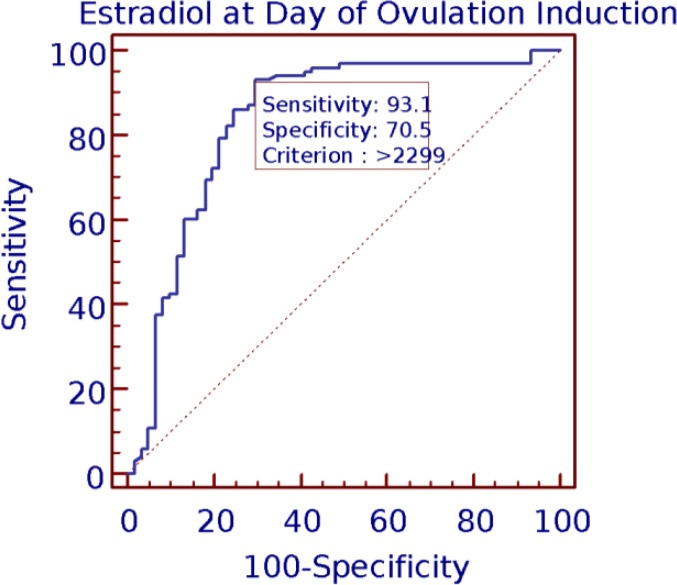
Estradiol at ovulation induction and clinical pregnancy by ROC curve

**Figure 4:**
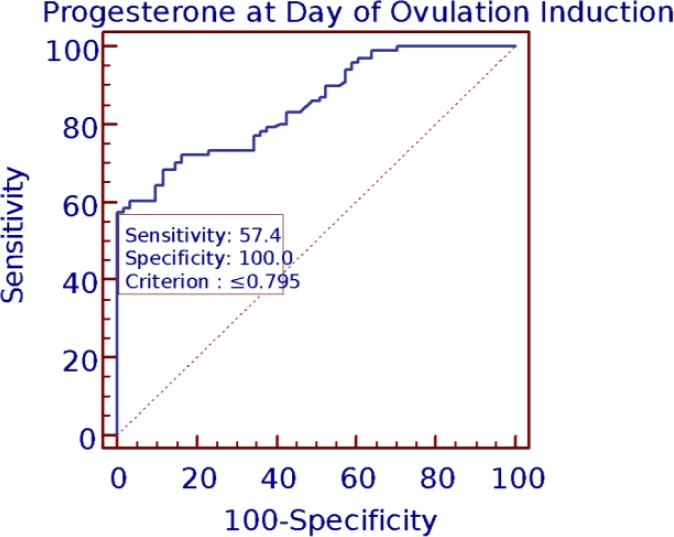
Progesterone at ovulation induction and clinical pregnancy ROC curve

**Figure 5:**
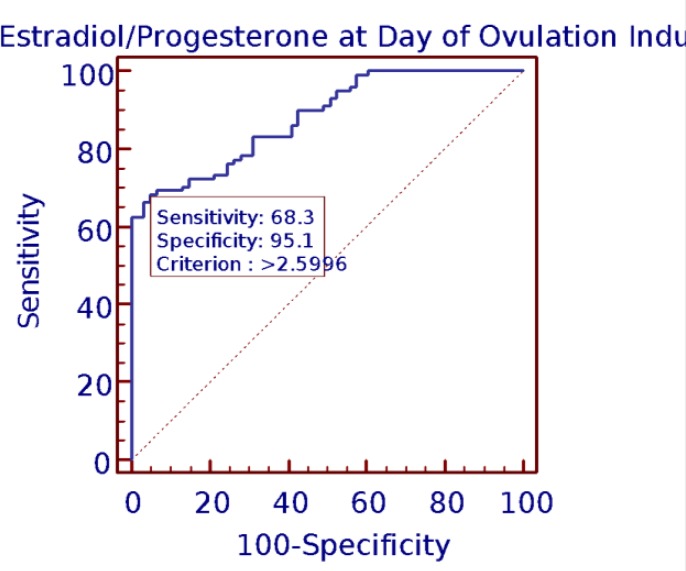
Estradiol Progesterone ratio and clinical pregnancy ROC curve

The best cut-off of E_2_ at day of OI was 2299 pg/ml. The best cut-offs for P on this day was 0.794 ng/ml. The values 2.59 was the best cut-offs for the ratio of E_2_/P at day of OI. To analyze the prognostic power of E_2_, P and E_2_/P ratio on OI with respect to clinical pregnancy, the AUC was determined with ROC analysis (Figure 3, 4, 5). The area under the curve on OI day suggests a prediction accuracy of CP rate with E_2_ (r=0.83; CI=0.76-0.88; p<0.001), P (r=0.85; CI=0.78-0.90; p<0.001) E_2_/P ratio (r=0.88; 95% CI=0.81-0.92; p=0.001) respectively. All the predictors accounted to produce significantly high accuracy levels with good values of sensitivity and specificity.

**Table I:** Study of hormones in outcome groups

**Hormones**	**Group I** **(Non pregnant) ** **(n=120)**	**Group II** **(Preclinical abortion) (n=61)**	**Group III** **(Clinical pregnancy) (n=101)**
E_2_/P ratio	1.26	1.62	3.96
Follicle stimulating hormone mIU/ml a	6.6 (1.54)	6.5 (1)	6.4 (0.7) d
Luteinizing hormone mIU/ml^a^	4.7 (1.75)	4.72 (1.15)	5.7 (1.62) ** d
Prolactin ng/ ml^a^	21.89 (6.89)	21.89 (8.66)	21.89 (6.32)
Basal estradiol pg/ml^a^	112.41 (30.6)	182 (176.33)	258.6 **^d^ (296.89)
Estradiol on ovulation induction pg/ml^c^	2322.5 (137.6)	2199 (238)	2488 (291) **^d^
Basal progesterone (ng/ml)^b^	8.26 (4.16)	11.76 (6.64)	14.8 (14.6) **^d^
Progesterone on ovulation induction (ng/ml)^c^	2.06 (1.04)	1.47 (0.83)	0.74 (0.73) d

Median (interquartile range) expressed.

a Estimation on second day of cycle.

b Estimation on day 21 of previous cycle

c Estimation on day of hCG administration

d Results of clinical pregnancy significant with non- pregnant; p<0.01

** Results of clinical pregnancy showed significant differences compared with preclinical abortion; p<0.01

**Table II:** Comparison of ovarian response to stimulation

**Hormones**	**Group I ** **(Non-pregnant) ** **(n=120)**	**Group II ** **(Preclinical abortion) (n=61)**	**Group III** **(Clinical pregnancy) (n=101)**	**p-value**
No of oocytes/patients	7 (2)	7 (4)	8 (2)	< 0.0001
No of oocytes metaphase II	6.5 (3)	7 (2)	8 (2)	< 0.0001
No of oocytes fertilized	5.5 (3)	6 (2)	7 (1)	< 0.0001
Number of puregons in one day	3.97 (0.91)	3.72 (0.75)	3.73 (0.61)	0.001
Total number of puregons	56.57 (10.97)	55.83 (6.78)	55.09 (5.15)	< 0.0001
Endometrial thickness	5 (6)	10 (4)	10 (5)	< 0.0001
Fertilization rate	83.33 (7.93)	83.33 (10.71)	83.33 (9.72)	0.203
Implantation rate	0 (0)	0 (0)	100 (50)	< 0.0001

Median (interquartile range) expressed.

## Discussion

Embryo implantation is a well-orchestrated series of events, which is aided by presentation of a receptive endocrine milieu created by secretion of specific hormones and regulation factors from functional corpus Luteum (19). These hormones and cytokines produced by trophoblastic cell cross the maternal-fetal interface and directly influence the systemic physiological changes which characterize implantation followed by conception. Successful implantation is attributed to availability of top quality embryos and receptive endometrium endorsed to optimal levels of hormones precisely E_2_ and P (19). 

The peak E_2_ aims to sustain optimal levels of P during the implantation period as well as in the luteal phase of female cycle. These hormones regulate locally produced cytokines, growth factors, home box transcription factors and cyclooxygenase-derived prostaglandins through autocrine and paracrine pathways (20). E_2_/P ratio is thus a supposed marker for endometrial receptivity which up regulates adhesion molecules on the endometrial pinopods and equivalent ligands on the blastocyst for successful implantation (16). 

Chorionic villi of developing embryo release hCG which was observed in 58% of the study patients in the form a positive beta hCG test whereas a higher E_2_/P ratio in 36% females made conception possible as was evident by fetal cardiac activity on TVS. Patients in this group had high E_2_/P ratio on OI day with elevated peak E_2_ levels as compared to those who had preclinical abortions or those who did not become pregnant at all. The positive association of higher peak E_2_ with ovarian and reproductive outcome observed in our study is comparable with findings in many studies (19, 21, 22). With high E_2_ peak fewer ampoules of rFSH were required meaning reduced dose for stimulation as was observed by others (23). 

The fact that elevated E_2_ peak increases the maturity of oocytes by increasing meiotic competency was noticed in our research (20). Increased retrieval of oocytes, availability of blastocysts and higher pregnancy rates with better peak E_2_ levels are comparable with few other studies (18, 24). Role of P as a key player in the commencement and continuation of pregnancy through complex endocrine and immune interactions has been well established (13, 25). The P level on the day of hCG administration has been used as an indicator of premature luteinization (PL) with cut off level from 0.8-2 ng /ml or ratio of P/E_2_ ratio greater than one (26, 27). 

High P peak in our study had deleterious effects on oocyte quality and endometrial secretory transformation that has been documented by others as early closure of implantation window ensuing failure of implantation. In our study a cut off value less than 0.794 was associated with pregnancy (28). Comparison of E_2_ and P in all the outcome groups affirmed that high E_2_/P ratio on the day of OI in group III (CP) correlated with increased number of retrieved, mature and fertilized oocytes. Few studies documented that moderately increased P with a high E_2_/P ratio is related to better pregnancy outcomes after ICSI. Gruber *et al* measured the E_2_/P ratio on fourth, fifth and seventh day after OI whereas Rehman *et al* assessed it seven days after OI (11, 15). 

Wessam *et al* determined a high E2/P ratio in CP group three days after OI but their results were not significant (16). The evaluation was done five days after ET by Souter *et al* and reported an insignificant higher E_2_/P ratio with unsuccessful cycles. The results of these studies are subject to dissimilarity on the basis of estimation after ET and luteal support by P supplementation (29). The correlation between BMI, reduced E_2_/P level and CP in present research is attributed to harmful effect of increased BMI on ovulatory function that reduces peak E_2_, endometrial proliferation and may even lead to termination of cycle (15, 30-32) The contradictory results of E_2_/P ratio measured on different days of cycle suggested the need to explore the estimation of ratio on OI before P supplementation. It is therefore the first study carried out in females to appraise the role of follicular hormones in the transformation of E_2_ prepared endometrium into a secretory tissue hence promote a conducive environment for embryo implantation after ICSI.

## Conclusion

The proportionate high levels of E_2_ with respect to P secreted by the oocytes in the follicular phase reflects better oocyte quality parameters and assists in increasing endometrial receptivity essential for successful pregnancy outcome after ICSI. 

## Conflict of interest

There is no conflict of interest in this research.

## Footnote

***The article extracted from Ph.D. thesis. (Rehana Rehman)***
